# Mean of the Typical Decoding Rates: A New Translation Efficiency Index Based on the Analysis of Ribosome Profiling Data

**DOI:** 10.1534/g3.114.015099

**Published:** 2014-12-01

**Authors:** Alexandra Dana, Tamir Tuller

**Affiliations:** * Department of Biomedical Engineering, Faculty of Engineering, Tel Aviv University, Ramat Aviv 69978, Israel; † Segol School, Tel Aviv University, Ramat Aviv 69978, Israel

**Keywords:** codon decoding rate, codon usage bias, gene translation, ribosome profiling, transcript evolution

## Abstract

Gene translation modeling and prediction is a fundamental problem that has numerous biomedical implementations. In this work we present a novel, user-friendly tool/index for calculating the mean of the typical decoding rates that enables predicting translation elongation efficiency of protein coding genes for different tissue types, developmental stages, and experimental conditions. The suggested translation efficiency index is based on the analysis of the organism’s ribosome profiling data. This index could be used for example to predict changes in translation elongation efficiency of lowly expressed genes that usually have relatively low and/or biased ribosomal densities and protein levels measurements, or can be used for example for predicting translation efficiency of new genetically engineered genes. We demonstrate the usability of this index via the analysis of six organisms in different tissues and developmental stages. Distributable cross platform application and guideline are available for download at: http://www.cs.tau.ac.il/~tamirtul/MTDR/MTDR_Install.html

Gene translation is a fundamental intracellular process. Thus, the ability to predict gene translation elongation efficiency (*i.e.*, a gene’s translation rate) is a central challenge related to all biomedical disciplines.

Currently, there are no direct measures of genes translation efficiency that do not include “components” of other gene expression stages, such as transcription and/or posttranslational regulatory steps (*e.g.*, mRNA degradation, protein degradation, and protein synthesis rate). For example, conventional nondirect proxies of translation efficiency include messenger RNA (mRNA) levels, protein abundance, or the normalization of the two aforementioned factors. One drawback of these proxies is the fact that they are not available for the majority of organisms in various experimental conditions and tissues. In addition, these proxies are not highly reliable for lowly expressed genes, nor they can predict the translation efficiency of new engineered genes expressed in the same cell conditions.

Recently, ribosome profiling was suggested for measuring some aspects of the translation process at nucleotide resolution (Ribo-seq) (Ingolia *et al.* 2009). This method can potentially estimate the relative time ribosomes spend on the organismal mRNA molecules, at nucleotide resolution. Thus, ribosome profiles reflect *in vivo* the translation process of specific tissues and developmental stages or conditions. As a result, it was suggested to estimate the general translation efficiency of genes by calculating their mean average footprint read counts (Ingolia *et al.* 2009).

However, resulting ribosome profiles are reliable only for highly expressed genes, thus restricting the ability of the method to accurately measure translation efficiency of the remaining of the genes or to predict translation efficiency of newly engineered genes in similar cellular conditions. For example, as can be seen in Figure 2, for *S. cerevisiae*, *H. sapiens*, and *M. musculus* only 13.9–23.7% of the genes include more than 50% positions with nonzero mapped read counts; similarly, only 8.5–11.8% of their genes include mean footprint count (FC; per nucleotide) larger than 2.

Additional conventional approach/indexes for estimating translation efficiency are based on various measures of codon distribution/bias within the opening reading frame (ORF) (Sharp and Li 1987; Wright 1990; dos Reis *et al.* 2004; Fox and Erill 2010; Sabi and Tuller 2014). These indexes were found to be correlative with the protein abundance in the cell for *S. cerevisiae*, *E. coli*, and *C. elegans* (dos Reis *et al.* 2004; Tuller *et al.* 2010b; Sabi and Tuller 2014). However, these indexes are not condition nor tissue specific and may not be directly related to translation but to other steps of gene expression and gene evolution (Sharp and Li 1987; Plotkin and Kudla 2011).

In contrast to the previous suggested indexes, the mean of the typical decoding rates (MTDR) index (Dana and Tuller 2014) is based on the estimation of the typical codon decoding times from Ribo-seq data, thus potentially capturing aspects of translation elongation in specific tissues, developmental stages, and/or conditions. Specifically, the MTDR index calculates the geometrical mean of the estimated typical nominal translation rates of a gene’s codons after filtering biases and phenomena such as ribosomal traffic jams and translational pauses (Dana and Tuller 2014) (see also Figure 1 and the section *Materials and Methods*), reflecting the mean typical translation elongation rate of a gene. Thus, this index could be used to predict all genes’ translation efficiency, including newly engineered genes.

**Figure 1:**
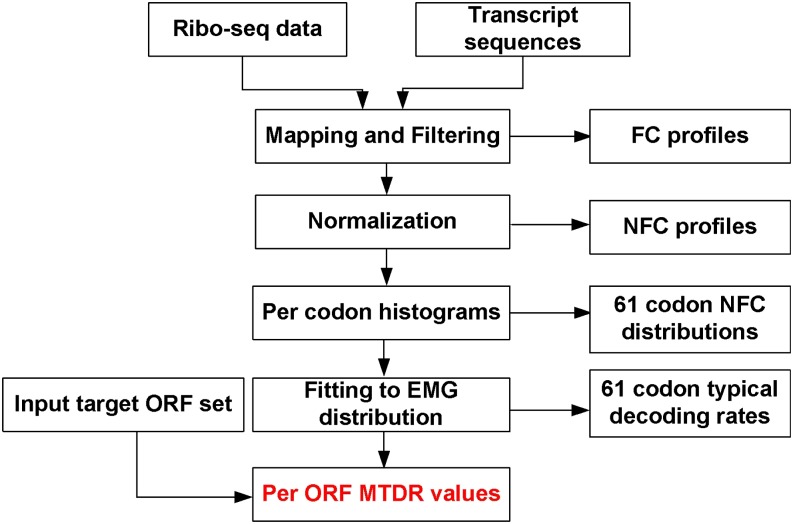
Diagram showing the mean of the typical decoding rates (MTDR) calculation process. Ribo-seq data are created per selected organism (condition and tissue specific). Resulting mRNA fragments are then mapped to transcript sequences resulting for each gene its ribosomal profile. Genes with mean footprint counts (FC) lower than one are filtered. To enable comparison between footprint counts of different genes with different mRNA levels and initiation rates, FC of each gene are normalized, resulting in normalized footprint count (NFC) profiles. Then for each codon type a NFC distribution is created. By fitting the NFC distribution to an exponentially modified Gaussian distribution type, the typical decoding rate of each codon is estimated. Finally, for each one of the input target open reading frames (ORFs), the mean typical decoding rates of its codons (MTDR index) is calculated.

In this study we estimate the typical codons translation time of additional organisms in different conditions and tissues (see Table 1) from ribosomal profiling data (see the section *Materials and Methods*). We also demonstrate the advantages of the MTDR index in predicting various gene expression measurements and compare it with previously suggested translation efficiency indexes (which are based on codon distributions). Finally, we provide a cross-platform tool for calculating the MTDR index of all ORFs in these organisms and conditions.

**Table 1:** Analyzed organisms and tissues/conditions

Organism	Condition/Tissue
*E. coli* (Li *et al.* 2012)	−
*B. subtilis* (Li *et al.* 2012)	−
*S. cerevisiae*	Exponential (Ingolia *et al.* 2009), (Brar *et al.* 2012)
	DNA replication (Brar *et al.* 2012)
	Recombination (Brar *et al.* 2012)
	Metaphase II (Brar *et al.* 2012)
	Anaphase (Brar *et al.* 2012)
	Spore packing (Brar *et al.* 2012)
	Spores (Brar *et al.* 2012)
*C. elegans* (Stadler *et al.* 2012)	L4 (Stadler *et al.* 2012)
	L2
	L1
*M. musculus*	Embryonic stem cells (Ingolia *et al.* 2011)
	Neutrophils (Guo *et al.* 2010)
	Embryonic fibroblast (Lee *et al.* 2012)
*H. sapiens*	HEK293 (Lee *et al.* 2012)

## Materials and Methods

### Calculating the normalized footprint count (NFC) distribution

**Figure 2:**
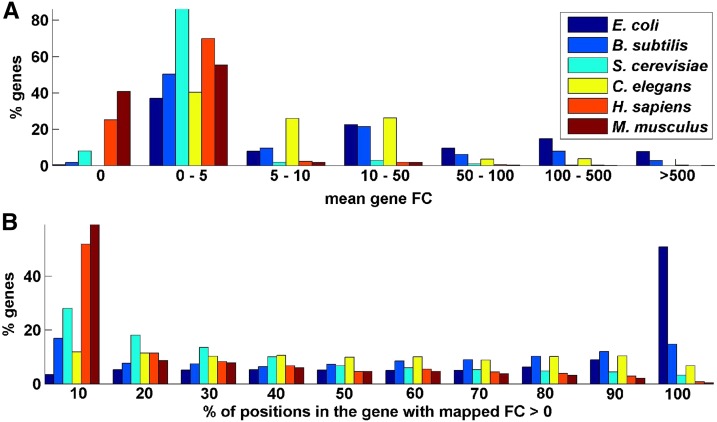
Footprint count (FC) statistics over all genes of six organisms. (A) Histogram of average read counts. (B) Histogram of percentage of positions with a positive number of mapped read counts. As can be seen in all analyzed organisms, most of the genes have very low read counts.

As seen in Figure 2, the majority of genes’ ribosome profiles have less than 50% of codons mapped with read counts. Therefore, to avoid analyzing unreliable ribosome profiles that could biases estimations, only genes with a median FC greater than one were included in the analysis (Dana and Tuller 2014). In addition, previous studies indicated an increase of FC at the beginning of the ORF (Ingolia 2010; Ingolia *et al.* 2011) and for some organisms at the end of ORF (Li *et al.* 2012); therefore, the first and last 20 codons were excluded from the analysis. Moreover, to prevent analysis of unreliable reads, codons with FC values less than one were excluded from the analysis (Li *et al.* 2012).

To enable comparison of footprint counts of a codon type from genes with different mRNA levels and initiation rates, FC of each codon were first normalized by the average FC of each gene (Li *et al.* 2012; Qian *et al.* 2012; Dana and Tuller 2014), resulting in NFC. This normalization enables measuring the relative time a ribosome spends translating each codon in a specific gene relative to other codons in it, while considering the total number of codons in the gene. Then, for each codon type a vector consisting of NFC values originating from all analyzed genes was generated, creating the “NFC distribution” of a codon.

### Estimating the codons’ typical decoding time

Based on the characteristics of the NFC distributions, we suggest that their topology could result from a superposition of two distributions/components (Dana and Tuller 2014): the first one describes the “typical” decoding time of the ribosomes, which was modeled by a normal distribution characterized by its mean μ and variance $\sigma^{2}$.

The second component describes relatively rare translational pauses and ribosomal interactions such as traffic jams due to the codons’ different translation efficiency and was modeled by a random variable with an exponentially distribution, characterized by one parameter λ.

The summation of two independent normal and exponential random variables corresponding to the distributions mentioned above results in an exponentially modified Gaussian distribution. The parameters μ,σ,λ were estimated by fitting the measured NFC distributions to the exponentially modified Gaussian distribution, under the log-likelihood criterion. The μ parameter is referred as the typical decoding time of a codon. For more details, see Dana and Tuller (2014). Then, the MTDR index of a gene was defined as the geometrical mean of its codons translation rates.

### Protein abundance and mRNA levels measurements

*E. coli* protein abundance measurements were downloaded from the PRIDE database (Vizcaino *et al.* 2013), and mRNA level measurements were taken from another source (Taniguchi *et al.* 2010). *C. elegans* mRNA levels were downloaded from Kirienko and Fay (2007). *B*. *subtilis* protein abundance measures were used as published by Chi *et al.* (2011), and mRNA levels were downloaded from another source (Nicolas *et al.* 2012). *S. cerevisiae* protein abundance measures were averaged from four quantitative large-scale measurements: two large scale measurements in two conditions (Newman *et al.* 2006), and a large-scale protein abundance measurement from two sources (Ghaemmaghami *et al.* 2003; Lee *et al.* 2011). mRNA levels were determined by averaging large scale measurements of mRNA levels (Wang *et al.* 2002; Ingolia *et al.* 2009).

### Estimating the contribution of the MTDR index and other translation efficiency indexes to predicting protein abundance

To estimate whether the MTDR index improves the prediction protein levels relative to other translation efficiency indexes, we have modeled the relationship between protein abundance and the different translation efficiency indexes MTDR, transfer RNA (tRNA) adaptation index [tAI (dos Reis and Wernisch 2009)], and codon adaptation index [CAI (Sharp and Li 1987)], using a linear regressor (Seber and Lee 2012).

$$\tilde{P}A=c+w_{1}MTDR+w_{2}tAI+w_{3}CAI$$

where the coefficients $c,w_{1},w_{2},w_{3}$ were estimated to minimize the mean square error difference between the prediction vector $\tilde{P}A$ and the real protein abundance measurements PA.

For each one of the coefficients $c,w_{1},w_{2},w_{3}$ confidence intervals (Kendall and Stuart 1979) were calculated to determine the reliability of the estimates (at 95% confidence interval). A coefficient’s confidence interval that does not contain zeroes implies that it significantly contributes to the regression, *i.e.*, the coefficient is not zero (Kendall and Stuart 1979).

## Results

### The estimated codons decoding times correlate with measures of codon usage bias

We started our analysis by comparing the typical decoding times estimated from the ribosome profiling data (µ) to other estimations of codon translation efficiency estimated using additional methods which are based on codon distributions. These include: 1) **d**ecoding **t**ime based on the **c**odon **a**daptation **i**ndex ($DT_{CAI}$), which calculates the codon bias in highly expressed genes (Sharp and Li 1987) and 2) **d**ecoding **t**ime based on the **t**RNA **a**daptation **i**ndex ($DT_{tAI}$) (dos Reis *et al.* 2004), which takes into consideration the number of tRNA copies in the genome recognizing each codon and additional codon/anticodon interactions. As seen in Table 2, the correlation between µ values and the other codon decoding time estimators is significant in all analyzed organisms (0.42 < *P* < 0.83; *P* < 0.00065).

**Table 2:** Spearman correlation between µ and various methods for estimating the typical codon decoding time

Estimation method	*E. coli*	*B. subtilis*	*S. cerevisiae*	*C. elegans*
$DT_{CAI}$	r = 0.55	r = 0.66	r = 0.56	r = 0.67
	*p* = 4*10^−6^	*p* = 8.8*10^−9^	*p* = 3.2*10^−6^	*p* = 4.3*10^−9^
$DT_{tAI}$	r = 0.42	r = 0.51	r = 0.47	r = 0.83
	*p* = 0.00065	*p* = 3.1*10^−115^	*p* = 0.00013	*p* = 1.6*10^−16^

In addition, we also compared the $\mu /DT_{CAI}/DT_{tAI}$ values to decoding times measured by using various experimental methods. For example, the amino acid insertion time per codon in *E. coli* was estimated using known biochemistry factors (Fluitt *et al.* 2007). Spearman correlation between insertion time and µ was 0.43 (*P* = 0.00051), whereas the correlation between insertion time and $DT_{tAI}/DT_{CAI}$ was lower: 0.35/0.31 (*P* = 0.0062/0.017), supporting the conjecture that µ values are better direct estimators of the amino acid insertion time. In another study (Chu *et al.* 2014) the authors found that for *S. cerevisiae* the GAG codon is more slowly translated than the GAA codon. This result is also supported by the µ estimations (GAG: 0.24 *vs.* GAA: 0.21). The same trend was also observed for the $DT_{CAI}$ values (GAG: 0.016 *vs.* GAA: 0.006) and $DT_{tAI}$ values (GAG: 2.44 *vs.* GAA: 1.1). In another study (Kemp *et al.* 2013) it was shown that in *S. cerevisiae*, replacing codons the codons “CAA” by the codons “CAG,” which are decoded by a rare tRNA, near the 5′ end of an ORF reduces luciferase expression by 60%. Indeed the estimated µ decoding time of the CAG codon was found to be greater than of the CAA codon (0.25 *vs.* 0.15). An additional work (Letzring *et al.* 2010) studied the effect of synonymous codon repeats of luciferase expression in *S. cerevisiae*; to validate our µ estimations, we calculated Spearman correlation between translation efficiency changes with respect to the wild type and the estimated µ values and found them to be significantly correlative (r = −0.33, *P* = 0.013).

### The MTDR index predicts protein levels in standard conditions with comparable quality as other codon bias measures

**Figure 3:**
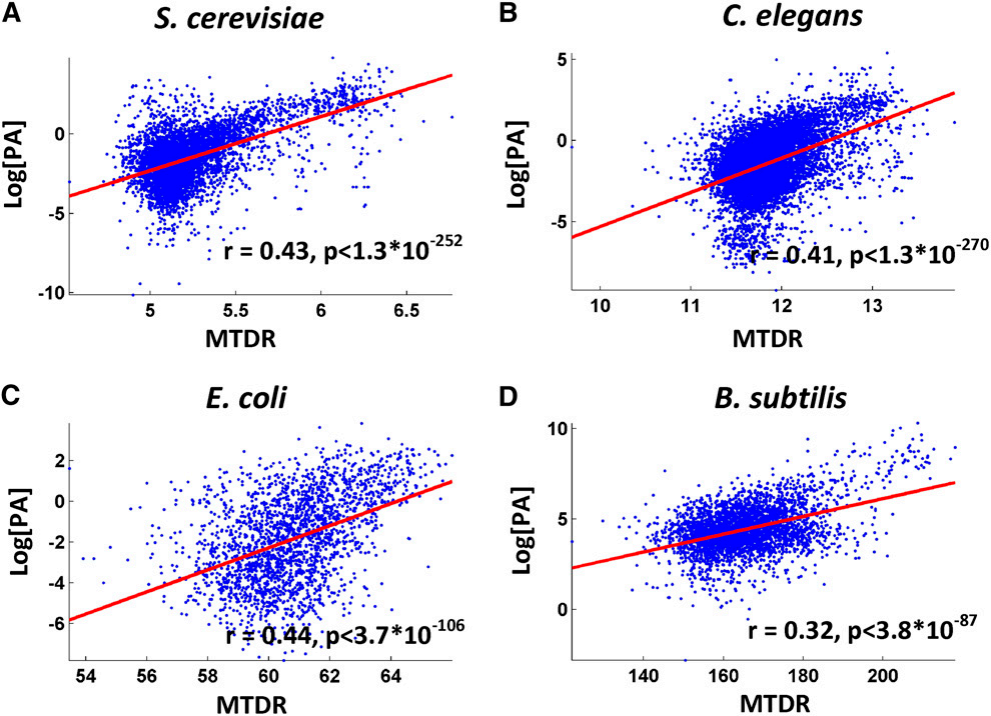
Protein abundance (PA) *vs.* mean of the typical decoding rates (MTDR) and Spearman correlation between MTDR index and protein abundance for various organisms.

The usability of the suggested MTDR index was validated for four different organisms that have abundant large scale protein and mRNA levels measurements (*E. coli*, *B. subtilis*, *C. elegans*, and *S. cerevisiae)*. Spearman correlation between the MTDR index to various proxies of translation efficiency such as protein abundance resulted in significant correlations (0.32< r < 0.44; *P* < 3.8*10^−87^; see also Figure 3 and Table 3). Similarly, a significant partial Spearman correlation between protein abundance and MTDR index given mRNA levels (partial correlation) was observed (0.23 < r < 0.5; *P* < 4.2*10^−86^; see Table 3).

**Table 3:** Spearman correlation between protein abundance (also when controlling for mRNA levels) and various translation efficiency indexes

Organism	Correlation Between Index and PA			Correlation Between Index and PA Given mRNA Levels		
	MTDR	CAI	tAI	MTDR	CAI	tAI
*E. coli*	r = 0.44	r = 0.56	r = 0.55	r = 0.5	r = 0.61	r = 0.48
	*p* < 3.7*10^−106^	*p* = 3.8*10^−184^	*p* =1.2*10^−178^	*p* < 1.1*10^−38^	*p* = 3.4*10^−62^	*p* = 3.4*10^−35^
*B. subtilis*	r = 0.32	0.37	r = 0.41	r = 0.26	r = 0.280	r = 0.32
	*p* < 3.8*10^−87^	*p* = 1.5*10^−117^	*p* = 4.7*10^−146^	*p* < 3.7*10^−43^	*p* = 7.1*10^−67^	*p* = 8.6*10^−108^
*C. elegans*	r = 0.41	r = 0.52	r = 0.56	r = 0.23	r = 0.35	r = 0.41
	*p* < 1*10^−270^	*p* = 0	*p* = 0	*p* < 4.2*10^−86^	*p* = 3.7*10^−206^	*p* = 6.1*10^−293^
*S. cerevisiae*	r = 0.43	r = 0.44	r = 0.47	r = 0.3	r = 0.33	r = 0.37
	*p* < 1.3*10^−252^	*p* = 1.1*10^−275^	*p* = 2.6**10^−314^	*p* < 1.2*10^−102^	*p* = 5.3*10^−125^	*p* = 1.1*10^−66^

PA, protein abundance; MDTR, mean of the typical decoding rates; CAI, codon adaptation index; tAI, tRNA adaptation index.

Measures based on codon usage bias resulted in similar correlations with protein levels in standard conditions (Table 3); these results can be explained among others, by the fact that codon bias usage measures various (direct and indirect) aspects of gene expression (Chamary *et al.* 2006; Hershberg and Petrov 2008; Plotkin and Kudla 2011; Sauna and Kimchi-Sarfaty 2011) (Tuller and Zur, unpublished data), by the fact that ribosome profiling data are probably “noisier” and more biased than genomic sequence data (Dana and Tuller 2012; Gerashchenko and Gladyshev 2014), and by the fact that in this study we consider protein levels in “standard”/exponential conditions which are probably reflected more properly by codon bias usage.

For some of the analyzed organisms, a decrease in correlation between the different translation efficiency indexes and protein abundance was observed when controlling for mRNA levels. As genes with greater mRNA levels potentially consume a greater percentage of the ribosomes in the cell, they are expected to undergo stronger selection forgreater translation elongation speed (and/or other aspects of translation efficiency) to reduce ribosome utilization (Tuller *et al.* 2010a; Plotkin and Kudla 2011). Therefore we expect to see a positive correlation between measures of translation efficiency and mRNA levels (and not only with protein abundance); as a result, the partial correlation between protein levels and translation efficiency indexes decreases when controlling for mRNA levels.

Because the correlation between the MTDR index and protein abundance was found to be similar to the correlation between protein abundance and the other two translation efficiency indexes, we wanted to assess whether the MTDR index provides *additional* information with respect to the other two. To this end, we calculated a linear regressor based on tAI, CAI, and MTDR for predicting protein abundance levels (see the section *Materials and Methods*). Then, we checked the 95% confidence intervals of each one of the translation efficiency indexes and found that they do not include the value 0 for the variable MTDR (see the section *Materials and Methods*), demonstrating that the MTDR index contributes additional information to protein abundance prediction given the other two indexes.

Finally, it should be noted that in endogenous genes it is impossible to prove the direction of causality between the various translation efficiency indexes and protein abundance based on correlation; a correlation may suggest that 1) codons with greater µ values or codons that are recognized by more abundant tRNA molecule tend to improve translation rate and thus increase protein levels (*e.g.*, Letzring *et al.* 2010; Tuller *et al.* 2010b); 2) genes with greater expression levels are selected to have codons with a greater MTDR/tAI index due to reasons not directly related, increasing the number of proteins per mRNA [for example, global ribosomal allocation (Kudla *et al.* 2009)].

### Using the MTDR index for predicting ribosomal load

**Figure 4:**
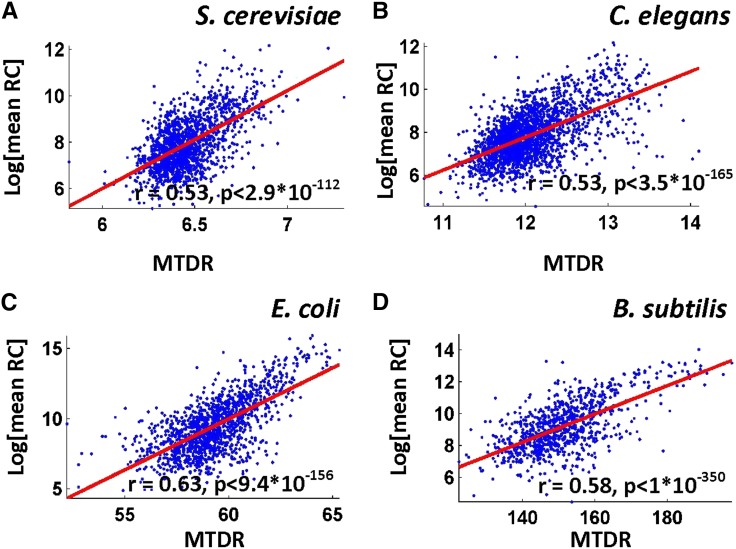
Ribosomal load, as estimated by the mean footprint counts per gene *vs.* mean of the typical decoding rates (MTDR) index for various organisms and Spearman correlation between these factors.

To demonstrate that the MTDR index can predict ribosomal densities of each one of the analyzed organisms we used 50% of the highly expressed genes to compute the typical codon decoding times. Next, we computed the MTDR index of the genes in the rest 50% of the highly expressed genes (that were not used for computing the typical decoding times). Finally, we computed the correlation between the MTDR index and the actual mean read count of these genes. As can be seen in Figure 4 and in Table 4, in almost all analyzed organisms/conditions the correlations are positive and significant (the top correlation is 0.8, *P* = 1.4*10^−131^). This result demonstrates that the MTDR index could be used as a good predictor of the ribosome load (number of ribosomes per mRNA * number of mRNA molecules), probably since highly translated genes tend to undergo selection for greater codon elongation rate, for example, to improve ribosomal allocation and translation cost (Kudla *et al.* 2009; Tuller *et al.* 2010a). For comparison, we also calculated the correlation between the tAI and CAI indexes and ribosomal load (see Table 4). These indexes resulted in similar correlations for the different “typical”/exponential stage conditions, however, produced a lower correlation in atypical conditions such as the different *S. cerevisiae* meiosis stages and for some *M. musculus* tissue types.

**Table 4:** Correlation between MTDR index and ribosomal load for the different tissues/conditions of the analyzed organisms

Organism	Correlation Between MTDR Index and Ribosomal Load	Correlation Between tAI Index and Ribosomal Load	Correlation between CAI Index and Ribosomal Load
*E. coli* (Li *et al.* 2012)	r = 0.63 *p* = 9.4*10^−156^	r = 0.56 *p* = 1.3*10^−117^	r = 0.60 *p* = 5.7*10^−142^
*B. subtilis* (Li *et al.* 2012)	r = 0.58 *p* = 0	r = 0.33 *p* = 0	r = 0.56 *p* = 0
*S. cerevisiae* – Exponential (Ingolia *et al.* 2009),	r = 0.53 *p* = 2.9*10^−112^	r = 0.62 *p* = 5.5*10^−163^	r = 0.65 *p* = 3*10^−184^
*S. cerevisiae* – Exponential (gb15) (Brar *et al.* 2012)	r = 0.32 *p* = 1*10^−22^	r = 0.29 *p* = 2.3*10^−18^	r = 0.32 *p* = 1.3*10^−22^
*S. cerevisiae* – Exponential (A14201) (Brar *et al.* 2012)	r = 0.80 *p* = 1.4**10^−131^	r = 0.77 *p* = 7.9*10^−116^	r = 0.80 *p* = 1.1*10^−134^
*S. cerevisiae* – DNA replication (Brar *et al.* 2012)	r = 0.26 *p* = 1.9*10^−8^	r = 0.11 *p* = 0.023	r = 0.09 *p* = 0.048
*S. cerevisiae* – Recombination (Brar *et al.* 2012)	r = 0.31 *p* = 2.4*10^−12^	r = 0.20 *p* = 7.6*10^−6^	r = 0.20 *p* = 1.4*10^−5^
*S. cerevisiae* – Metaphase II (Brar *et al.* 2012)	r = 0.46 *p* = 4.3*10^−8^	r = -0.12 *p* = 0.15	r = -0.12 *p* = 0.18
*S. cerevisiae* – Anaphase (Brar *et al.* 2012)	r = 0.23 *p* = 0.027	r = 0.10 *p* = 0.31	r = 0.11 *p* = 0.26
*S. cerevisiae* – Spore packing (Brar *et al.* 2012)	r = 0.50 *p* = 0	r = -0.15 *p* = 0.045	r = -0.16 *p* = 0.029
*S. cerevisiae* – Spores (Brar *et al.* 2012)	r = 0.35 *p* = 0.00015	r = -0.11 *p* = 0.25	r = -0.08 *p* = 0.39
*C. elegans* – L4 (Stadler *et al.* 2012)	r = 0.53 *p* = 3.5*10^−165^	r = 0.45 *p* = 1.8*10^−112^	r = 0.49 *p* = 1.9*10^−132^
*C. elegans* – L2 (Stadler *et al.* 2012)	r = 0.48 *p* = 1.6*10^−197^	r = 0.47 *p* = 1.4*10^−185^	r = 0.51 *p* = 1.4*10^−219^
*C. elegans* – L1 (Stadler *et al.* 2012)	r = 0.51 *p* = 5.9*10^−10^	r = 0.42 *p* = 2.8*10^−72^	r = 0.46 *p* = 9.3*10^−86^
*M. musculus* – Embryonic stem cells(Ingolia *et al.* 2011)	r = 0.02 *p* = 0.35	r = 0.07 *p* = 0.0095	r = 0.07 *p* = 0.0046
*M. musculus* – Neutrophils (Guo *et al.* 2010)	r = 0.22 *p* = 1.2*10^−13^	r = 0.07 *p* = 0.012	r = 0.12 *p* = 5.4*10^−5^
*M. musculus* – Embryonic fibroblast (Lee *et al.* 2012)	r = 0.41 *p* = 4.1*10^−15^	r = 0.22 *p* = 3.6*10^−5^	r = 0.22 *p* = 3.4*10^−5^
*H. sapiens* – HEK293 (Lee *et al.* 2012)	r = 0.17 *p* = 1.9*10^−9^	r = 0.15 *p* = 9.2*10^−8^	r = 0.15 *p* = 3.4*10^−8^

MDTR, mean of the typical decoding rates; tAI, tRNA adaptation index; CAI, codon adaptation index.

**Figure 5:**
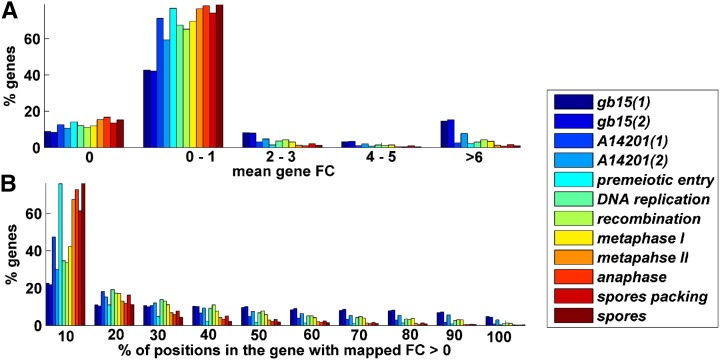
Footprint count (FC) statistics for *S. cerevisiae* different meiosis stages. (A) Histogram of average read counts. (B) Histogram of percentage of positions with a positive number of mapped read counts. As can be seen in all of the organisms, most of the genes have very low read counts.

It should be mentioned that a decrease in this correlation was observed for non-exponential stages in *S. cerevisiae* and in greater eukaryotes such as *H. sapiens* and *M musculus*. This could result from: 1) a greater level of noise (see Figure 5) and biases caused by additional and/or more complicated biological mechanisms (*e.g.*, due substantial alternative splicing in mammals the mapping of reads to exons is less trivial) (Engstrom *et al.* 2013); 2) the fact that mammals have smaller effective population size and thus lower selection pressure related to some translation aspects (Charlesworth 2009; dos Reis and Wernisch 2009); and 3) since growth rate is strongly related to fitness in unicellular organisms but not in mammals, there is lower effect on ribosomal allocation on the organisms fitness in mammals than in the rest of the analyzed organism (Rocha 2004; dos Reis and Wernisch 2009).

### Assessing the ability of the MTDR index to predict translation efficiency in different experimental conditions

Codon bias indexes (*e.g.*, CAI, tAI) are based solely on static information encoded in the genome or general chemical properties; thus, they cannot differentiate among different experimental conditions. One of the major advantages of the MTDR index is that it is condition specific. To demonstrate this advantage, we estimated the typical decoding times of *S. cerevisiae* in starvation conditions (Ingolia *et al.* 2009). Translation efficiency (defined as protein abundance normalized by mRNA levels) was calculated for rich (yeast extract peptone dextrose) and minimal (synthetic defined) media [protein abundance and mRNA levels measured per cell were taken from a previous study (Newman *et al.* 2006)].

**Figure 6:**
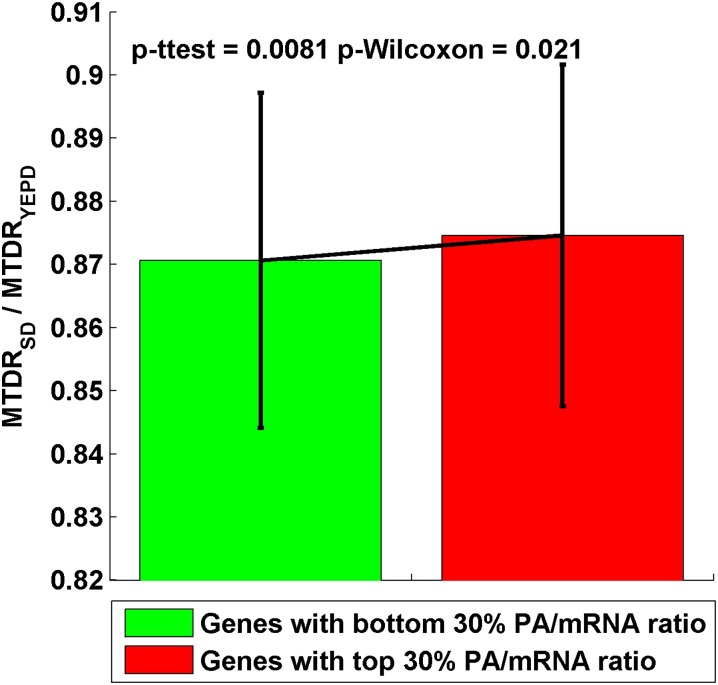
The ration between mean of the typical decoding rates (MTDR) index calculated in starvation (synthetic defined [SD]) and rich medium (yeast extract peptone dextrose [YEPD]) for genes with bottom/up 30% PA/mRNA ratio in SD/YEPD conditions (green/red).

We found that the MTDR values of the genes with top/bottom 30% translation efficiency ratios are significantly different (*t*-test: *P* = 0.0081; Wilcoxon test: *P* = 0.021): genes with a greater change in their translation efficiency have a greater change in their estimated MTDR index (in the same direction). This result demonstrates the ability of the suggested index to estimate gene expression in different conditions (Figure 6).

In addition, we also found that Spearman correlation between the MTDR ratio and ribosomal load ratio for *different tissue/developmental conditions* in *C. elegans*, *S. cerevisiae*, and *M. musculus* (Table 5) was significantly positive, (0.17 < r < 0.72; *P* < 0.0003), indicating that the MTDR index could predict changes in ribosomal load, contrary to the static tAI and CAI indexes.

**Table 5:** Spearman correlation between MTDR index ratios and ribosomal load ratio for different tissues/developmental stages in *C. elegans*, *S. cerevisiae* (partial list) and *M. musculus*

Compared Conditions	Corr(MTDR1/MTDR2, meanRC1/meanRC2)	Compared Conditions	Corr(MTDR1/MTDR2, meanRC1/meanRC2)
*C. elegans* L4 *vs.* L2	r = 0.53 *P* = 0	*S. cerevisiae* premeiotic entry *vs.* metaphase I	r = 0.40 *P* = 0.00045
*C. elegans* L4 *vs.* L1	r = 0.51 *P* = 4.5*10^−52^	*S. cerevisiae* premeiotic entry *vs.* spore packing	r = 0.33 *P* = 0.044
*C. elegans* L2 *vs.* L1	r = 0.51 *P* = 0	*S. cerevisiae* premeiotic entry *vs.* spores	r = 0.56 *P* = 0.00042
*S. cerevisiae* gb15 *vs.* A14201	r = 0.59 *P* = 5.8*10^−8^	*S. cerevisiae* DNA replication *vs.* metaphase II	r = 0.44 *P* = 0.014
*S. cerevisiae* gb15 *vs.* premeiotic entry	r = 0.42 *P* = 0.00011	*S. cerevisiae* recombination *vs.* metaphase II	r = 0.54 *P* = 3.7*10^−5^
*S. cerevisiae* gb15 *vs.* recombination	r = 0.32 *P* = 2*10^−6^	*S. cerevisiae* metaphase I *vs.* metaphase II	r = 0.45 *P* = 6.7*10^−5^
*S. cerevisiae* gb15 *vs.* metaphase II	r = 0.55 *P* = 5.2*10^−6^	*S. cerevisiae* metaphase II *vs.* anaphase	r = 0.45 *P* = 0.0031
*S. cerevisiae* gb15 *vs.* spore packing	r = 0.34 *P* = 0.00074	*S. cerevisiae* metaphase II *vs.* spore packing	r = 0.38 *P* = 0.055
*S. cerevisiae* gb15 *vs.* spores	r = 0.45 *P* = 0.0013	*S. cerevisiae* spore packing *vs.* spores	r = 0.52 *P* = 0.00079
*S. cerevisiae* A14201 *vs.* premeiotic entry	r = 0.72 *P* = 0.0031	*M. musculus* embryonic stem cells *vs.* neutrophils	r = 0.17 *P* = 0.0003
*S. cerevisiae* A14201 *vs.* recombination	r = 0.56 *P* = 6.5*10^−5^	*M. musculus* embryonic stem cells *vs.* fibroblast	r = 0.25 *P* = 0.00074
*S. cerevisiae* A14201 *vs.* spore packing	r = 0.56 *P* = 0.0096	*M. musculus* neutrophils *vs.* fibroblast	r = 0.31 *P* = 0.00023

## Application

The MTDR application enables calculating the translation efficiency of various genes according to their ORF sequence. The input of the application includes the selection of one of the organisms and its tissue/condition and a file containing the ORFs of the requested genes (in FASTA format or text format where each ORF is defined in a separate line). ORFs could also be straightforwardly inserted in a textbox. The application returns an output file which includes for each of the ORFs their MTDR index. The available organisms and conditions/tissues are depicted in Table 1. The distributable cross platform application and user manual are available for download at:

http://www.cs.tau.ac.il/~tamirtul/MTDR/MTDR_Install.html
